# Consistent Differential Expression Pattern (CDEP) on microarray to identify genes related to metastatic behavior

**DOI:** 10.1186/1471-2105-12-438

**Published:** 2011-11-11

**Authors:** Lam C Tsoi, Tingting Qin, Elizabeth H Slate, W Jim Zheng

**Affiliations:** 1 Bioinformatics Graduate Program, Department of Biochemistry and Molecular Biology, Medical University of South Carolina, 135 Cannon St. Charleston, SC 29425, USA; 2 Department of Statistics, Florida State University, 117 N. Woodward Ave. Tallahassee, FL 32306, USA; 3 Division of Bioinformatics, Department of Biochemistry and Molecular Biology, Medical University of South Carolina, 135 Cannon St. Charleston, SC 29425, USA

## Abstract

**Background:**

To utilize the large volume of gene expression information generated from different microarray experiments, several meta-analysis techniques have been developed. Despite these efforts, there remain significant challenges to effectively increasing the statistical power and decreasing the Type I error rate while pooling the heterogeneous datasets from public resources. The objective of this study is to develop a novel meta-analysis approach, Consistent Differential Expression Pattern (CDEP), to identify genes with common differential expression patterns across different datasets.

**Results:**

We combined False Discovery Rate (FDR) estimation and the non-parametric RankProd approach to estimate the Type I error rate in each microarray dataset of the meta-analysis. These Type I error rates from all datasets were then used to identify genes with common differential expression patterns. Our simulation study showed that CDEP achieved higher statistical power and maintained low Type I error rate when compared with two recently proposed meta-analysis approaches. We applied CDEP to analyze microarray data from different laboratories that compared transcription profiles between metastatic and primary cancer of different types. Many genes identified as differentially expressed consistently across different cancer types are in pathways related to metastatic behavior, such as ECM-receptor interaction, focal adhesion, and blood vessel development. We also identified novel genes such as *AMIGO2*, *Gem*, and *CXCL11 *that have not been shown to associate with, but may play roles in, metastasis.

**Conclusions:**

CDEP is a flexible approach that borrows information from each dataset in a meta-analysis in order to identify genes being differentially expressed consistently. We have shown that CDEP can gain higher statistical power than other existing approaches under a variety of settings considered in the simulation study, suggesting its robustness and insensitivity to data variation commonly associated with microarray experiments.

**Availability**: CDEP is implemented in R and freely available at: http://genomebioinfo.musc.edu/CDEP/

**Contact**: zhengw@musc.edu

## Background

Investigating transcription profile by microarray technology has been one of the most promising genomic approaches in the last decade. Thousands of microarray experiments were performed for this purpose and their data made available through databases such as Gene Expression Omnibus, ArrayExpress and Stanford Microarray Database [1-3]. To utilize this massive amount of information, investigators have developed different meta-analysis techniques--parametric approaches such as t-statistic [4,5]; Fisher's inverse Chi-square approach [6]; Bayesian [7-9], and non-parametric approaches [10,11]. However, these approaches still face many challenges in combining data from different sources [12,13]. For example, parametric Bayesian models used in meta-analysis [7,8] are not appropriate due to the small sample size for many datasets, as suggested by Kong et al. [11]. On the other hand, non-parametric methods such as RankProd-based meta-analysis approach (Meta-RankProd) [14,15] can be significantly influenced by the size of the dataset and hence biased toward genes that are only differentially expressed in a dataset with a large number of samples--an undesirable outcome for studies where the objective is to find genes with differentially expressed patterns common across the datasets. The method of Rhodes et al. [16], which we refer to as Meta-Profile, combines a parametric and non-parametric approach and gives equal weight to each dataset when counting the number of times a gene is identified as differentially expressed in all datasets. However, this is a significant simplification because the resulting power to identify differentially expressed genes and Type I error rate (i.e. false positive rate) vary by dataset according to sample size and proportion of genes truly differentially expressed [17]. The challenges faced by these methods are particularly evident when identifying genes differentially expressed across different cancer types by pooling datasets from various sources. These datasets typically have small sample sizes [18] and the analyses are influenced by cancer-type and/or cancer-subtype specific effects [16,19,20]. In addition, some methods such as Meta-RankProd do not handle varying numbers of differentially expressed genes from different datasets--an issue that needs to be addressed for a meta-analysis approach to be robust.

The objective of this study is to develop a robust meta-analysis approach to identify genes with consistent differential expression patterns across different datasets. In our study, we combined FDR and the non-parametric RankProd approach to estimate the Type I error rate in each dataset. The estimated rates from all datasets were combined using a Bernoulli likelihood to identify genes with common expression pattern. The robustness of this approach in obtaining high statistical power was shown by simulation studies. We then applied the method to analyze different microarray data that compared gene expressions between metastatic and primary cancers and identified a core gene set that is critical to cancer metastasis across different cancer types. Our analysis identified many genes annotated in pathways that are related to metastasis, as well as novel genes that have not been shown to associate with, but may play roles in, metastasis. Further sensitivity analysis indicates that the method is robust and can be applied to other datasets for similar analyses.

## Results

### Consistent differential expression pattern (CDEP)

**Figure 1:**
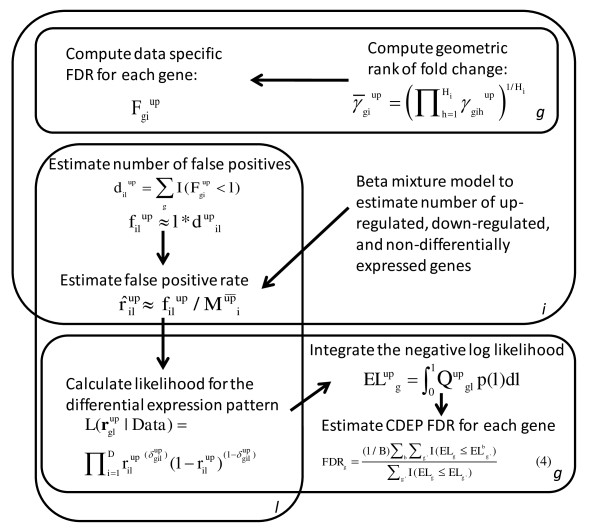
**The workflow of CDEP**. Each "plate" in the figure represents an instance of gene (*g = 1,2,...G*), dataset (*i = 1,2,...D*), or dataset local FDR threshold (*l *∈ *(0,1)*). This workflow diagram only illustrates how to identify consistently up-regulated genes, but the same procedure was applied to identify consistently down-regulated genes.

The key components of CDEP are the application of: 1) consistent FDR across datasets to identify significant genes [16,21]; and 2) non-parametric rank product (RankProd) approach to identify differentially expressed genes from microarray experiments [10]. By first using a consistent FDR to estimate the Type I error rates in each dataset, CDEP avoids overemphasizing datasets with large sample sizes--a drawback of a previous RankProd-based meta-analysis approach (Meta-RankProd) [14]. CDEP then uses the error rates from all datasets to identify genes with consistent differential expression patterns. Figure 1 shows the workflow of CDEP.

Specifically, let dataset *i, i = 1, 2, ..., D*, consist of gene expression levels for *m_i _*and *n_i _*samples in each of two conditions, respectively (e.g. *m_i _*cases and *n_i _*controls). For dataset *i*, the geometric mean rank of gene *g *= 1, 2, ..., G was computed across all *m_i_n_i _*= *H_i _*pairwise comparisons for up-regulation:

(1a) $${{\bar{\gamma }}_{gi}}^{up}={\left(\prod_{h=1}^{H_{i}}{\gamma_{gih}}^{up}\right)}^{1∕H_{i}}$$

and down-regulation:

(1b) $${{\bar{\gamma }}_{gi}}^{down}={\left(\prod_{h=1}^{H_{i}}{\gamma_{gih}}^{down}\right)}^{1∕H_{i}}$$

where γ*_gih _*is the rank of fold change for gene *g *in the *h^th ^*comparison of dataset *i*, *h = 1, 2, ..., H_i_*. Genes with the smallest RankProd values (${\bar{\gamma }}_{gi}$) are more likely to be the differentially expressed genes.

We then computed the RankProd p-values and FDRs for up- and down-regulations for each gene in every dataset [10]. Briefly, we used permutation of the sample labels (e.g. case/control) to estimate false positives and the p-value by counting the number of times we observed the permutations' RankProd values smaller or equal to the experiment's RankProd value. The FDR of a gene was then estimated by dividing the p-value by the rank of the RankProd value [10]. Each gene in every dataset was thus associated with an FDR, ${F_{gi}}^{up}$(${F_{gi}}^{down}$), for being up(down)-regulated. For genes not present in the platform of a dataset, the median FDR value computed for that dataset was assigned.

This computation in CDEP was performed using the Bioconductor [22] package RankProd [15], as Hong and Breitling [14] indicated that RankProd is more reliable than other existing approaches (see also Additional File 1, Figure S1). The FDR threshold (*l*) is defined as the proportion of false positives among the genes declared to be positives for each dataset. Given an FDR threshold (*l*), we counted the number of genes identified to be

up-regulated:

(2a) $${d_{il}}^{up}=\underset{g}{\sum }I\left(F_{gi}^{up}<l\right)$$

and down-regulated:

(2b) $$\;{d_{il}}^{down}=\underset{g}{\sum }I\left(F_{gi}^{down}<l\right)$$

**Figure 2:**
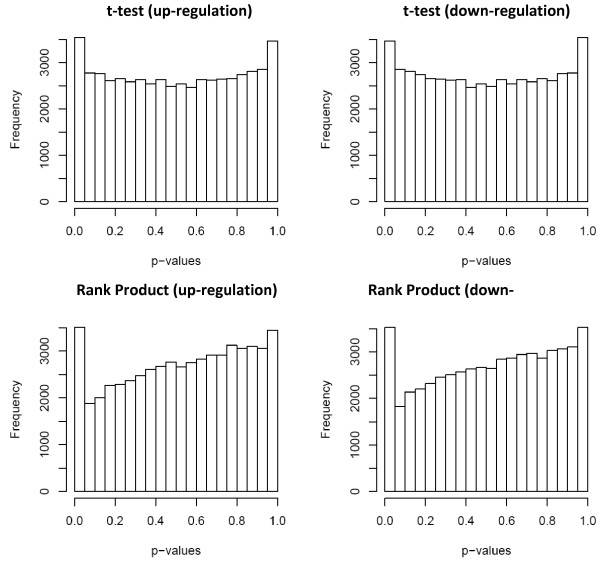
**The distributions of p-values computed by t-test and RankProd test**. We compared the parametric t-test and the non-parametric rank product approaches to test which of these p-value (computed from one-sided test) calculation is robust for identifying truly differentially expressed genes (p = q = 0.05; |Δ| = 0.5).

Therefore, the number of false positives in a dataset could be estimated as ${f_{il}}^{up}\approx l*{d^{up}}_{il}$(${f_{il}}^{down}\approx l*{d^{down}}_{il}$). To estimate the proportion of genes that are up-regulated, non-differentially expressed, and down-regulated, we used a Beta mixture to model the genes' p-values for over (under)-expression in each dataset (see details of the Beta mixture model in Methods). We adopted the Beta mixture model because the p-values calculated from our non-parametric approach do not have a uniform distribution for non-differentially expressed genes (Figure 2), in contrast to a previous mixture model based on this assumption [23]. The Beta mixture model and the estimation of the proportion of differentially expressed genes used the Markov Chain Monte Carlo (MCMC) technique implemented in the BUGS program [24]. Our implementation uses WinBUGS [25] on the Windows platform, but OpenBUGS [26] can be used on Linux or Mac platforms (with Wine).

For each dataset, the false positive rate is defined as the probability of a non-up-regulated (non-down-regulated) gene being falsely called as over-expressed (under-expressed): ${{\hat{r}}^{up}}_{il}\approx {f_{il}}^{up}∕{M^{ū\bar{p}}}_{i}$$\left({{\hat{r}}^{down}}_{il}\;\approx {f_{il}}^{down}∕{M^{\bar{down}}}_{i}\right)$, where ${M^{\bar{up}}}_{i}$ (${M^{\bar{down}}}_{i}$) are the number of genes that are not up(down)-regulated in dataset *i *respectively, estimated by the Beta mixture model. Based on this rate and using independent Bernoulli distributions, we calculated the likelihood of a gene to be falsely identified as over or under-expressed among the datasets for each FDR threshold *l*, that is, the likelihood for false positives among the significant genes identified as

up-regulated:

(3a) $$L(r_{gl}^{up}|Data)=\prod_{i=1}^{D}r_{il}^{up}{}^{\;(\delta_{gil}^{up})}{\left(1-r_{il}^{up}\right)}^{\left(1-\delta_{gil}^{up}\right)}$$

and down-regulated:

(3b) $$L(r_{gl}^{down}|Data)=\prod_{i=1}^{D}r_{il}^{down}{}^{\;(\delta_{gil}^{down})}{\left(1-r_{il}^{down}\right)}^{\left(1-\delta_{gil}^{down}\right)}$$

where the binary variable $\delta_{gil}^{up}$ (or $\delta_{gil}^{down}$) indicates whether gene *g *is identified to be up(or down)-regulated in dataset *i *for threshold *l*. To prevent underflow during computation, we worked with the minus log likelihood, *Q*, e.g. for up-regulation ${Q^{up}}_{gl}=-\ln\left[L\left(r_{gl}^{up}|Data\right)\right]$. We took into consideration of multiple FDR thresholds *l *by specifying a probability density function (PDF) for *l*, *p(l)*, *l*∈(0, 1) and using the expected value of *Q(l) *to assess whether the gene is consistently over-expressed among the datasets. In this assessment, low *l *values were emphasized because low FDR represents a higher proportion of true positives, and we used the linear decreasing function: *p*(*l*) = -2*l *+ 2. The expected log likelihood across the FDR threshold is: $E{L^{up}}_{g}=\int_{0}^{1}Q_{gl}^{up}p\left(l\right)dl$, which was approximated by discretizing the range of FDR value (*l*) into one hundred bins with equal width and using the rectangular rule. The same procedure was also performed for down-regulation. The procedure was evaluated by estimating the false discovery rate (*FDR_g_*) of observing the above expected log likelihood. Here, the *FDR_g _*is the proportion of false positives among the genes identified to be consistently differentially expressed. The "null log likelihood" was computed by permuting the ${F_{gi}}^{up}$ and ${F_{gi}}^{down}$ values relative to the genes within each dataset and performing the same above procedures to calculate the expected value of the "null log likelihood" in each permutation *b *for every gene ($E{L^{b}}_{g}$). In *b *permutations, the *FDR_g _*of a gene could be determined as:

(4) $$FDR_{g}=\frac{\left(1∕B\right)\sum_{b}\sum_{g^{\prime }}I\left(EL_{g}\leq E{L^{b}}_{g^{\prime }}\right)}{\sum_{g^{\prime }}I\left(EL_{g}\leq EL_{g^{\prime }}\right)}$$

**Figure 3:**
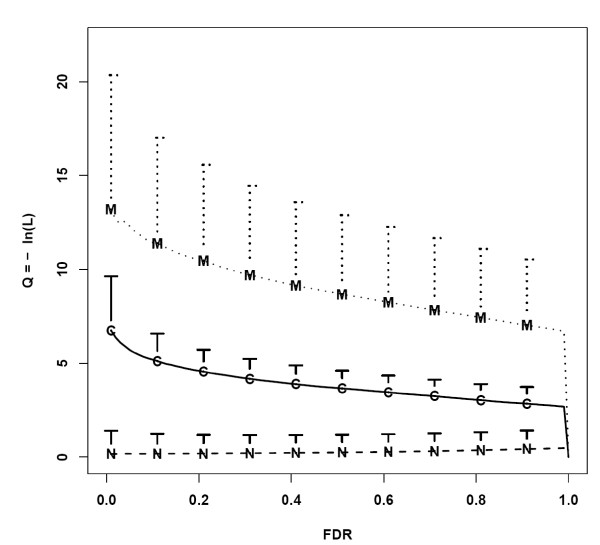
**Log likelihood and FDR plot**. Minus log likelihood versus the FDR threshold (*l*) for different genes in one of the simulated data (proportion of cancer-type specific and metastatic related differentially expressed genes: *p *= *q *= 0.1; degree of effect: |Δ| = 1). FDR is the proportion of false positives among genes declared to be differentially expressed for each dataset. The dotted line represents genes that are consistently differentially expressed, solid line represents genes that are differentialy expressed only in specific dataset, and dashed line represents non-differentially expressed genes. The three lines show the mean, and the vertical bars show the standard deviation of the Q values for the three types of genes at the given FDR. For clarity, only the upper bars are shown.

**Figure 4:**
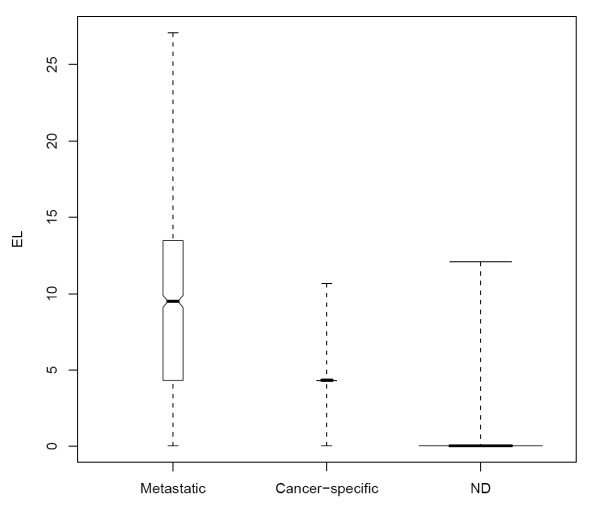
**Boxplots for the expected likelihood**. The boxplots for the expected likelihood (EL) of the three categories of genes: genes that are consistently differentially expressed, genes differentially expressed in only a certain dataset, non-differentially expressed genes. The ranges and the quartiles are shown. The width of the boxplot is drawn proportional to the square-root of the number of observations. p = q = 0.1, |Δ| = 1.

The robustness of CDEP in distinguishing different gene expression patterns is shown in Figure 3 where the minus log likelihood value *Q *was plotted against *l*. Genes that are not differentially expressed in all datasets (*G_N_*) have the lowest *Q *values, while genes that are differentially expressed only in some datasets (*G_C_*) have higher *Q *values, and genes that are differentially expressed in all the datasets (*G_M_*) have the highest *Q *values. For *G_N_*, the *Q *values increase slightly with *l*. This is because when *l *increases, the likelihood value decreases as more *G_N _*are falsely called differentially expressed. Moreover, even at high *l *many *G_N _*are not declared differentially expressed. On the other hand, the *Q *values of both *G_C _*and *G_M _*decrease when *l *increases. As *l *increases, *r *and the likelihood (*L*) increase, giving rise to a decreasing *Q*. Note that the *Q *values for all 3 types of genes go to zero when *l *= 1. This is because, in this situation, all genes in the array would be declared as differentially expressed and both *r *and *L *have values of one. Figure 4 shows the expected minus log likelihood (*EL*) for the three types of genes, indicating CDEP is robust in identifying genes that show common differential expression pattern across different datasets. These genes have higher *EL *values than the other two types of genes.

### Comparisons with other approaches

We compared CDEP with Meta-Profile and Meta-RankProd in a simulation study. Briefly, Meta-Profile is based on the number of times a gene is declared differentially expressed among the datasets, and Meta-RankProd uses the rank product among all datasets. Both approaches use permutation to estimate the false discovery rate for the genes as being differentially expressed consistently among the datasets. Simulation scenarios were determined by three key parameters: the proportion of differentially expressed genes that are dataset-specific (*p*), the proportion of genes that are consistently differently expressed (*q*), and the mean difference between the expression values in the two conditions being compared (Δ) (See "Simulation of Microarray data" in Methods and Simulation Section in Additional File 1 for details). Table 1 reports the statistical power and Type I error rate of the three meta-analysis approaches, where statistical power is defined as the sensitivity of detecting genes that have consistent differential expression patterns across datasets. The results show that raising FDR increases the statistical power and Type I error rate for all three approaches. Increasing the mean difference (|Δ|) between the two conditions (e.g. case vs. control) of the differentially expressed genes also improves sensitivity. In addition, the impact of the proportion of *G_M _*on CDEP and Meta-RankProd is obvious: the higher the proportion of *G_M _*(i.e., *q*), the lower the statistical power and Type I error rate. The reason is that obtaining FDR for these two approaches requires permutation and recalculation of $E{L_{g}}^{b}$ and $RP_{g}^{b}$. After permutation, original *G_M _*genes would act as *G_C _*in CDEP and *G_N _*in Meta-RankProd. As a result, when there is a higher proportion of *G_M _*from the datasets, including these genes to estimate FDR would potentially lead to over-estimation because the variance of these genes is different from the non-differentially expressed genes [27]. Therefore, under the same FDR, the statistical power and the Type I error would be lower for higher *q *in CDEP and Meta-RankProd, especially when comparing *q *= 0.1 with *q *= 0.2. In contrast, Meta-Profile takes a relatively conservative approach, and is insensitive to genes that do not have consistent differential expression patterns. However, the tradeoff is the loss in statistical power. As shown in Table 1, even though the Type I error rate is amongst the lowest of the three approaches, the Meta-Profile method has the lowest statistical power. Overall, CDEP emerges as a robust meta-analysis method that obtains comparably high statistical power while maintaining low Type I error rate under different simulated conditions (see Additional File 1, Section 3: Comparison Between Different Approaches for Genes Appearing in Different Numbers of Datasets. More simulation results can be found in Additional File 2).

**Table 1:** The Power and Type I error of CDEP, Meta-Profile and Meta-RankProd from simulation study.

				CDEP		Meta-Profile		Meta-RankProd	
*p*	*q*	|Δ|	FDR	Power (%)	Type I error	Power (%)	Type I error	Power (%)	Type I error
**0.05**	**0.05**	**1**	**0.05**	28.7	1.64 × 10^-4^	6.40	1.92 × 10^-6^	23.7	1.21 × 10^-2^
			**0.1**	31.0	3.52 × 10^-4^	9.10	1.92 × 10^-6^	24.5	1.23 × 10^-2^
			**0.2**	70.2	2.21 × 10^-3^	11.9	1.35 × 10^-5^	27.2	1.27 × 10^-2^
		**2**	**0.05**	33.4	2.02 × 10^-4^	15.4	1.35 × 10^-5^	33.2	1.18 × 10^-2^
			**0.1**	34.3	4.22 × 10^-4^	15.6	1.73 × 10^-5^	45.0	1.24 × 10^-2^
			**0.2**	74.9	2.28 × 10^-3^	18.2	2.31 × 10^-5^	56.3	1.38 × 10^-2^
**0.1**	**0.1**	**1**	**0.05**	26.2	2.29 × 10^-4^	8.93	2.84 × 10^-6^	23.6	2.29 × 10^-2^
			**0.1**	27.8	5.31 × 10^-4^	11.4	4.88 × 10^-6^	24.4	2.31 × 10^-2^
			**0.2**	33.1	1.84 × 10^-3^	13.4	1.12 × 10^-4^	26.7	2.36 × 10^-2^
		**2**	**0.05**	32.8	2.99 × 10^-4^	15.6	5.90 × 10^-5^	27.0	2.34 × 10^-2^
			**0.1**	33.1	6.02 × 10^-4^	18.2	1.16 × 10^-4^	32.2	2.39 × 10^-2^
			**0.2**	36.8	2.08 × 10^-3^	23.6	2.32 × 10^-4^	46.4	2.56 × 10^-2^
**0.05**	**0.1**	**1**	**0.05**	28.2	1.61 × 10^-4^	8.00	8.12 × 10^-6^	24.3	1.16 × 10^-2^
			**0.1**	29.5	3.11 × 10^-4^	10.7	1.83 × 10^-5^	25.7	1.18 × 10^-2^
			**0.2**	66.5	1.78 × 10^-3^	13.0	2.64 × 10^-5^	29.7	1.22 × 10^-2^
**0.1**	**0.2**	**1**	**0.05**	21.8	9.36 × 10^-5^	10.3	4.35 × 10^-5^	23.3	2.33 × 10^-2^
			**0.1**	26.3	4.68 × 10^-4^	12.5	9.14 × 10^-5^	23.5	2.34 × 10^-2^
			**0.2**	31.7	1.61 × 10^-3^	14.0	1.94 × 10^-4^	24.3	2.36 × 10^-2^

*p *is the proportion of genes differentially expressed only in a certain dataset, and *q *is the proportion of consistently differentially expressed genes; Δ is the simulated mean difference between the expression values in case and control condition for the differentially expressed genes. FDR is the proportion of false positives among the genes identified to be consistently differentially expressed across all datasets. The results in the table are the mean values of 10 different simulated datasets. Additional simulation results can be found in Additional File 2.

### Using CDEP to identify a core gene set that is differentially expressed in Metastatic cancer

We used CDEP to investigate the hypothesis that there exists a core gene set differentially expressed consistently in different types of metastatic cancer cells. Six different types of cancer were investigated for this purpose (Table 2) [28-34]. Totally there are 220 samples, of which 126 are from primary and 84 from metastatic cancer, respectively. The diversity of these datasets (i.e. a wide variety of labs, different numbers of samples and probesets for different experiments, etc.) make them ideal for assessing the robustness of CDEP and exploring our hypothesis. We used RMA [35] to pre-process the raw data for each dataset, and the median expression value of the probesets matching to the same Entrez gene id was used as the expression level for the gene.

**Table 2:** Description of the six microarray datasets used.

Cancer Type	Number of samples	Number of Metastatic samples	Affymetrix Platform	Number of probesets	Number of genes
Cervical	33	12	HG-U133 P2	5,4675	20,271
Prostate	90	25	HG-U95Av2	1,2625	9,000
Gastric	22	15	Hu6800	7,129	5,526
Colon	6	3	HG-U133A	22,283	13,069
OSCC*	27	19	HG-U133A	22,283	13,069
RCC^#^	32	10	HG-U133A	22,283	13,069

Raw data were downloaded from the NCBI GEO database. *OSCC = oral squamous cell carcinoma; ^#^RCC = renal cell carcinoma

At FDR ≤ 0.05, CDEP identified 239 genes that are differentially expressed consistently between the primary and metastatic cancer conditions across different cancer types. Out of these 239 genes, 141 were up-regulated and 98 down-regulated (Additional File 3). Table 3 shows the 5 most significantly up- and down-regulated genes identified. Using the same FDR criterion, we also performed meta-analysis by Meta-Profile and Meta-RankProd. Both CDEP and Meta-RankProd recovered the same two significant genes (*BSG and SLC25A1*) identified by Meta-Profile, and 180 genes were identified by both CDEP and Meta-RankProd (Meta-RankProd identified 2,967 significant genes. See Additional File 1, Figure S7 for details). A list of these genes identified by the three methods can be found in Additional File 4. These results further support using CDEP for meta-analysis to select candidate genes: Meta-Profile has insufficient statistical power, and Meta-RankProd tends to have high false positive rates. On the other hand, CDEP has the advantages of maintaining statistical power and keeping low false positive rates for identifying genes that are differentially expressed consistently.

**Table 3:** Five most significant genes identified by CDEP as related to common metastatic mechanism across different cancer types by using FDR < 0.05 as threshold

Up-regulated genes	Down-regulated genes
Glycoprotein (transmembrane) nmb (*GPNMB*)	Serpin peptidase inhibitor, clade B (ovalbumin), member 5 (*SERPINB5*)
Secreted phosphoprotein 1 (*SPP1*)	proteasome (prosome, macropain) subunit, beta type, 9 (*PSMB9*)
Transforming growth factor, beta-induced (*TGFBI*)	myxovirus (influenza virus) resistance 1, interferon-inducible protein p78 (*MX1*)
Heat shock 27kDa protein 1 (*HSPB1*)	interferon-induced protein with tetratricopeptide repeats 1 (*IFIT1*)
Mesoderm specific transcript homolog (*MEST*)	ubiquitin D (*UBD*)

The functional annotation of the 239 genes identified by CDEP is consistent with previous findings that many of them are involved in cancer metastasis. For instance, *GPNMB *overexpresses in a breast cancer cell line that could aggressively metastasize to bone [36], and *SPP1*'s expression level (also called Osteopontin) is elevated in a variety of metastatic cancers [37], including colon cancer [38], hepatocellular carcinoma [39], and gastric cancer [40]. Among the down-regulated genes, the expression level of *SERPINB5 *is negatively associated with the depth of invasion, metastasis, and TNM stage in gastric cancer [41]. Interestingly, *SERPINB5 *also inhibits invasion and metastasis of epithelial ovarian cancer, which suggests its down-regulation could promote metastasis [42]. *MX1 *was also predicted to have an inhibitory effect on tumor cell motility and invasion, an essential attribute for metastatic behavior. While most of these previous findings are specific to different cancer types, analysis from CDEP indicates that these genes could play important roles in metastatic mechanism common to all types of cancers.

**Figure 5:**
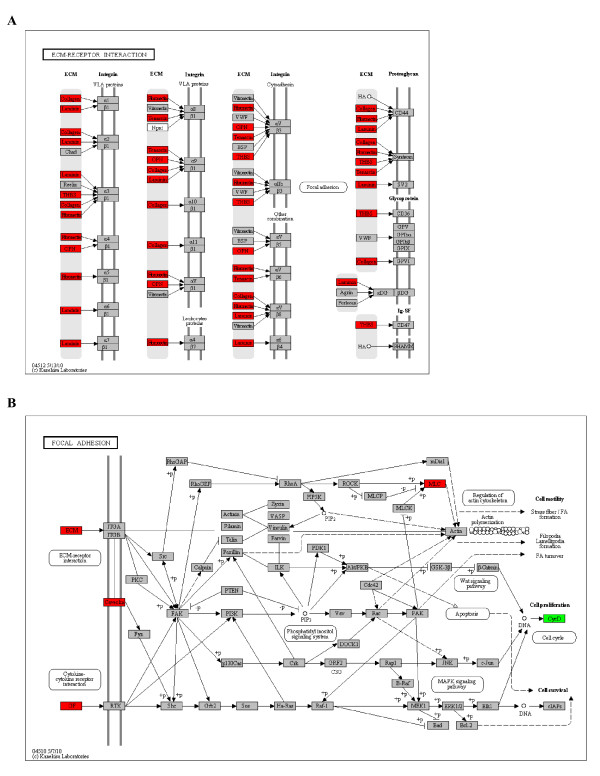
**CDEP identified differentially expressed genes map to biological pathways relevant to cancer metastasis**. A) ECM-receptor interaction pathway. B) Focal adhesion pathway. Up-regulated genes are annotated with red color, and down-regulated genes are in green.

The function of these 239 genes were further investigated by DAVID [43] through Gene Set Enrichment Analysis, using all genes present in the microarray platform as background. The results indicate that the up-regulated genes in metastatic cancer cells are enriched in extracellular matrix (ECM) receptor interaction, focal adhesion, and angiogenesis, while down-regulated genes are enriched in genes functioning in immune and inflammatory response (Table 4), and these include laminin, fibronectin, collagen, multimerin, caveolin, etc.. Figure 5 shows the CDEP identified genes mapped to the ECM receptor interaction and focal adhesion pathways. It is widely recognized that these pathways contribute to the aggressiveness and the metastatic behavior of cancer cells [44].

**Table 4:** Gene Set Enrichment Analysis to identify functional groups from CDEP identified genes.

Functions (source)	Annotated genes	FDR
ECM-receptor interaction (KEGG)	*COL4A2,COL4A1,TNC,COL3A1,COL5A2,COL5A1,COL6A3,COL1A2,COL1A1,LAMB1,COL11A1,THBS2*, *FN1,SPP1*	1.84 × 10^-8^
Focal adhesion (KEGG)	*CAV1,COL4A2,COL4A1,TNC,COL3A1,COL5A2,COL5A1,MYL9,VEGFC,VEGFA,COL6A3,COL1A2*, *COL1A1,LAMB1,THBS2,COL11A1,FN1,SPP1*	2.02 × 10^-7^
Blood vessel development (GO)	*PLAT,CAV1,IL8,COL3A1,COL15A1,COL5A1,VEGFC,APOB,APOE,CTGF,VEGFA,COL1A2,SEMA3C,LOX,COL1A1,CYR61*	1.10 × 10^-5^
Immune response (GO)	*CXCL1,POU2AF1,CCL2,BST2,CXCL3,IGJ,CXCL2,CXCL9,IL32,IFI44L,CCL5,CXCL11,HLA-DMA*, *HLA-DQA1,PSMB8,CXCL10,PSMB9,CXCL13,TAP1,DEFB1,GBP1*	2.06 × 10^-6^
Inflammatory response (GO)	*CXCL1,CCL2,NMI,CXCL3,CXCL2,CXCL9,ANXA1,IDO1,CXCL11,CCL5,CXCL10,FOS,SAA2,CXCL13*	5.95 × 10^-5^

The functional enrichment for the genes identified as consistently differentially expressed between primary and metastatic cancers.

Not only had CDEP identified genes known to be involved in cancer metastasis, but also it discovered novel genes that have not been implicated in metastatic mechanism. For example, *AMIGO2 *, a gene identified as differentially expressed by only CDEP out of the three meta-analysis approaches, is involved in anti-apoptosis [45] and cell adhesion [46], and CDEP shows that this gene is up-regulated under metastases conditions. *Gem *is also differentially expressed consistently in metastatic cancer cells, even though no current literature indicates its role in metastasis. However, the gene interacts with microtubule network [47] and regulates actin dynamics [48]. Such activities are highly related to the migratory and invasive properties of cancer metastasis [49]. Another gene, *CXCL11*, was shown to be consistently down-regulated by CDEP analysis. Given that this gene has angiostatic property [50], our results suggest that its down-regulation in metastatic cancer might interrupt the angiostasis process and promote angiogenesis--an important aspect of cancer metastasis.

## Discussion

Meta-analysis provides a cost-efficient way to approach biological problems. However, the heterogeneous nature of the data is always a significant challenge. CDEP aims to overcome this hurdle to identify genes that have a common differential expression pattern across different datasets. We illustrated that CDEP can: (i) obtain higher statistical power than existing meta-analysis approaches while maintaining low Type I error rate in the simulation study, and (ii) identify genes that are potentially involved in common metastatic behaviors and relevant biological pathways. CDEP borrows information from each dataset to identify genes differentially expressed consistently--a flexible approach that can be generalized to problems other than metastasis. The high statistical power under diverse sets of parameters considered in the simulation study also suggests robustness of CDEP to the diversity of data sources.

In CDEP, the minus log likelihood *Q *for different FDR values (*l*) was used because CDEP does not intend to "filter out" genes in each dataset before performing meta-analysis. This is in contrast to Meta-Profile where genes that only met the threshold (*l*) in each dataset were used for the meta-analysis. In CDEP, we emphasized low *l *values to calculate *EL *and thus employed a linearly decreasing PDF for the log likelihood to: 1) balance the "filtering" behavior that would result from a convex decreasing PDF; and 2) emphasize small *l*. The PDF used in CDEP outperformed Meta-Profile and Meta-RankProd in obtaining high statistical power and lowering Type I error rate.

CDEP, Meta-Profile, and Meta-RankProd use different permutation approaches to estimate *FDR_g_*. Meta-RankProd permutes gene expression values relative to the gene ID for each array while Meta-Profile and CDEP permute FDR relative to gene ID for each dataset examined. The null distribution produced by Meta-RankProd permutation would lead to *RP_g _*representing genes that are non-differentially expressed in any dataset, while Meta-Profile and CDEP would simply increase the proportion of genes that are differentially expressed in only a single dataset after permutation. Therefore, Meta-RankProd tends to under-estimate *FDR_g_*, as it ignores genes that are only differentially expressed in a single dataset. On the other hand, Meta-Profile and CDEP would over-estimate *FDR_g _*because they have a higher proportion of *G_C _*genes in the null distribution compared to Meta-RankProd. Even though inaccurate estimation of *FDR_g _*is inevitable due to the lack of prior knowledge about the proportion of genes only differentially expressed in one versus multiple datasets, both CDEP and Meta-Profile employed a more conserved approach than Meta-RankProd to obtain high precision.

## Conclusion

CDEP is a flexible meta-analysis approach that borrows information from each dataset in order to identify genes that are consistently differentially expressed. CDEP obtains higher statistical power than two existing approaches under a variety of scenarios considered in the simulation study, suggesting its robustness and insensitivity to data variation. By application to metastatic cancer datasets as a case study, CDEP allows identification of genes differentially expressed consistently in different types of metastatic cancer cells. These identified genes could be further developed into universal biomarkers for cancer staging and diagnosis.

## Methods

### Microarray data

We searched the published microarray datasets comparing primary and metastatic cancer samples from the NCBI Gene Expression Omnibus (GEO) database (http://www.ncbi.nlm.nih.gov/gds/). To ensure high quality, the included datasets and the associated publications needed to have clear descriptions about primary and metastatic cancer samples, and only single channel oligonucleotide array from Affymetrix was considered. In addition, we selected only datasets that provide raw data so we could apply the same pre-processing method for consistency. Since the goal of this study was to identify common genes across different cancer types, only one dataset from each cancer type was used to avoid biases. If a cancer type had multiple datasets that met our criteria, the dataset with the most precise and detailed description of the samples was included.

### Simulation of Microarray data

We used simulation to evaluate the performance of CDEP. Simulated data were generated to mimic the real experimental datasets retrieved. Specifically, the retrieved raw datasets listed in Table 2 were pre-processed using the Robust Multichip Average (RMA) algorithm [35] to generate the summarized probe readings of the probesets in logarithmic values. The resulting probesets were then matched to the corresponding NCBI Entrez gene id using the annotations from Affymetrix [51]. Because each gene will be matched to multiple probesets, the median expression value of these matched probesets was used for each gene. Overall, for each of the six different cancer types we selected, we generated a simulated dataset for meta-analysis. Each simulated dataset represented comparison of metastatic and primary cancer cells for the corresponding cancer type. The simulation was done using a modified version of a model described in Stevens and Doerge [52]:

(5) $$\begin{array}{lll} y_{gijk}= & \mu +L_{i}+G_{gi}+\alpha_{gi}\left(T_{gjk}\right)\; & \text{(1)}\\ & +\beta_{gij}\left(M_{gijk}\right)+\varepsilon_{gijk}\; & \text{(2)}\\ & \; & \text{(3)} \end{array}$$

where *y_gijk _*is the expression value of gene *g *in experiment *k *conducted by laboratory *i *in cancer condition *j *(*j *= 1 for metastatic; *j *= 0 for primary), *μ *is the universal background reading, and *L_i _*and *G_gi _*are the effects of laboratory *i *and gene *g *from laboratory *i*, respectively. We also incorporated binary variables *α_gj _*and *β_gij _*to distinguish genes that have common differential expression pattern from genes that are differentially expressed pertinent only to a specific dataset: differentially expression of gene *g *owing to the cancer-type specific effect is indicated by *α_gj _*= 1, and owing to the mechanism of the common metastatic behavior across different cancer types is indicated by *β_gij _*= 1. These indicators are used in the model to determine the contributions of cancer type specific and metastatic effects (*T_gik _*and *M_gijk_*, respectively) to the gene expression value. The detail for the simulation model is as followed:

$$\begin{array}{c} \mu =a_{1}\\ L_{i}\sim N\left(0,b_{2}\right)\\ G_{gi}\sim N\left(0,b_{3i}\right)\\ \alpha_{g1}\sim Bern\left(p\right)\\ \beta_{gi1}\sim Bern\left(q\right)\\ T_{gjk}\sim N\left(\Delta ,\psi \right),\text{ where }\Delta \neq 0\\ M_{gijk}\sim N\left(\Delta ,\psi \right),\text{ where }\Delta \neq 0\\ \varepsilon_{gijk}\sim N\left(0,e_{i}\right) \end{array}$$

In the simulation, we assumed that different datasets have different numbers of probesets and experiments. In each dataset, genes selected to be involved in cancer-specific effect (i.e. $\alpha_{g1}=1$) were randomly assigned as up- or down-regulated to make such behavior dependent on dataset (cancer type). Genes selected to be involved in the metastatic behavior common to all cancer type were also randomly assigned as up- or down-regulated genes to make it independent of dataset (cancer type). However, a gene could only be in at most one of these two categories, i.e. we required that $\alpha_{g1}+\beta_{gi1}$ is contained in the set {0, 1}. The above simulation parameters were estimated from microarray datasets comparing primary versus metastatic cancer cells (Table 2). During simulation, we used different values for the proportion of cancer-type specific (*p*) and metastatic related differentially expressed genes (*q*). We also examined different cancer-specific and metastatic-related effects Δ. We then applied CDEP to identify genes involved in metastatic behavior in this simulation study.

### Bayesian mixture model for p-value

We used a mixture of beta distributions to model the p-values arising from the RankProd method. Because the p-values correspond to genes that are up-regulated, non-differentially expressed, or down-regulated, we used a 3-component mixture model. The p-value for gene *g*, *y_g_*, is represented as $y_{g}=\overset{3}{\underset{k=1}{\sum }}T_{gk}p_{k},$where *T_g _= *(*T_g1_, T_g2_, T_g3_*) ~ Multinomial(θ, 1) so that exactly one element of *T_g _*is one and the remaining elements are zero. The value *p_k _*arises from the *k*^th ^component of the mixture: *p_k _*~ Beta(*a_k_*, *b_k_*), *k *= 1, 2, 3. We used a Dirichlet prior for θ, θ ~ Dir(1, 18, 1) and further assigned prior distributions as *a_1 _*= *b_3 _*= 1; *a_2 _*~ Gamma(4, 2); *a_3_, b_1 _*~ Gamma(400, 20), and *b_2 _*~ Gamma(1, 1), where the Gamma(α, β) is parameterized so that the mean is α/β.

### Comparison with other approaches

To assess the robustness of CDEP, we compared it with Meta-Profile and Meta-RankProd [14-16,21]. Meta-Profile is one of the pioneering methods to investigate common cancer signatures at large scale. This approach first identifies a dataset-specific "differential expression signature"--a list of differentially expressed genes for each dataset determined by the pre-defined threshold of FDR (*l*) [5]. The number of signatures each gene appeared in is then counted and permutation is performed to estimate the false positives of this count. The Meta-RankProd approach is a relatively recent approach that uses the rank product to identify genes differentially expressed between two conditions from multiple datasets. In this method, the rank fold change, *γ_gih_*, is computed as the ranking of gene *g *in the *h^th ^*comparison in the *i^th ^*study, and the rank product for gene *g *was calculated as the geometric mean across all comparisons. The null rank product was obtained by permuting expression values within each single array. This method was shown to outperform both the parametric t-based modeling approach [53] and the Fisher's inverse Chi-square approach [6] in terms of sensitivity and specificity. CDEP, Meta-Profile and Meta-RankProd were applied to analyze the simulated datasets to evaluate their performances in terms of: i) the statistical power to identify genes with common differential expression pattern across datasets; and ii) Type I error rate of falsely identifying genes without common differential expression. In this analysis, we tested the effect of different proportions of differentially expressed genes attributed to cancer-type specific (*p*) and metastatic-related (*q*). We also examined the effects of the detectable difference (Δ) of differential expression. For RankProd and CDEP, genes absent from a dataset were assigned the median rank value of that dataset.

## List of abbreviations used

**Table:** 

Symbol	Range	Annotation
*d_il_*	1,2,...,*G*	Number of genes in a dataset with FDR lower than the threshold *l *
*EL_g_*	(0,Inf)	Expected value of the log likelihood with respect to the FDR threshold
*f_il_*	1,2,...,*d_il_*	Number of false positives using the FDR threshold *l*
*F_gi_*	(0,1)	Gene-specific false discovery rate in dataset *i*: proportion of false positives among the significant calls
*FDR_g_*	(0,1)	Gene-specific false discovery rate for having consistently differentially expressed patterns among the datasets studied
*g*	1,2,...,*G*	Index for a gene from the union of gene sets across all datasets
*h*	1,2,...,*H_i_*	Index for fold change comparison between a case and a control from a dataset, where m_i _* *n_i _= H_i_*
*i*	1,2,...,*D*	Index for a gene expression microarray dataset (consists of *m_i _*cases and *n_i _*controls)
*l*	(0,1)	FDR threshold used to enumerate number of genes with FDR lower than this threshold in a dataset and to estimate the number of false positives under this threshold
*L*(***r****_gl_*|*Data*)	(0,1)	Gene- and FDR threshold- specific likelihood of observing the differential expressed pattern among the datasets
${M^{\bar{up}}}_{i}\left({M^{\bar{down}}}_{i}\right)$	1,2,...,*G*	number of genes that are not up(not down)-regulated in dataset *i*
*Q_gl_*	(0,Inf)	Minus log likelihood
${{\hat{r}}^{up}}_{il}({{\hat{r}}^{down}}_{il}\text{ )}$	(0,1)	False positive rate: the probability of a non-up-regulated (non-down-regulated) gene being falsely called as over-expressed (under-expressed)
$\delta_{gil}^{up}$ (or $\delta_{gil}^{down}$)	0[1]	Binary variable indicating gene *g *is identified as up(or down)-regulated in dataset *i *for threshold *l*
*γ_gih_*	1,2,...,*G*	rank of fold change for gene *g *in the *h^th ^*comparison of dataset *i*

## Conflict of interests

The authors declare that they have no competing interests.

## Authors' contributions

WJZ conceived the initial idea of the project and worked with LCT on data selection and analysis. EHS advised the statistical method development of the project. LCT and TQ wrote the R and Winbugs codes for the analysis. LCT drafted and WJZ and EHS finalized the manuscript. WJZ supervised the overall development of the project. All authors have read and approved the manuscript.

## Supplementary Material

Additional file 1 **Supplementary materials for the analysis**. Detailed descriptions about: 1) Datasets Used (Suppl. Table 1); 2) The comparisons between p-values computed by the parametric t-test versus the non-parametric RankProd (Suppl. Figure 1); 3) The Bayesian mixture for the p-value distribution (Suppl. Figure 2, Table 2 and Table 3); 4) Comparisons of different approaches for handling genes appearing in different numbers of datasets based on simulation (Suppl. Figure 3, Figure 4, Figure 5 and 6); and 5) Comparisons of the three approaches using the 6 cancer datasets as case study (Suppl. Figure 7). Click here for file

Additional file 2 **Results from the simulation data**. The statistical power and Type I error rate are compared for the three meta-analysis approaches on simulation data. Click here for file

Additional file 3 **Metastases-related genes identified by CDEP**. Statistically significant genes identified by CDEP as related to metastatic behavior by using *FDR *= *0.05*. Click here for file

Additional file 4 **Comparison of Significant Genes Identified by CDEP, Meta-Profile and Meta-RankProd**. List of genes that are differentially expressed consistently in metastatic cancer cells as identified by CDEP, Meta-Profile and Meta-RankProd from six data sets used. Click here for file
