# Supplementary material for: Consistent Differential Expression Pattern (CDEP) on microarray to identify genes related to metastatic behavior
Source: BMC Bioinformatics. 2011 Nov 11;12:438. doi: 10.1186/1471-2105-12-438 (PMC3251006; doi:10.1186/1471-2105-12-438)
Supplement: Additional file 1 — Supplementary materials for the analysis. Detailed descriptions about: 1) Datasets Used (Suppl. Table 1); 2) The comparisons between p-values computed by the parametric t-test versus the non-parametric RankProd (Suppl. Figure 1); 3) The Bayesian mixture for the p-value distribution (Suppl. Figure 2, Table 2 and Table 3); 4) Comparisons of different approaches for handling genes appearing in different numbers of datasets based on simulation (Suppl. Figure 3, Figure 4, Figure 5 and 6); and 5) Comparisons of the three approaches using the 6 cancer datasets as case study (Suppl. Figure 7). [file 1471-2105-12-438-S1.DOC]

**Supplementary Materials**

1. **Datasets Used**

We only used epithelial cell related cancers:

**Supplementary Table 1**: Metadata of the microarray samples

| Cancer | # of samples | # of probesets | mean |
| --- | --- | --- | --- |
| Cervical cancer | 33 | 54675 | 4.5123 |
| Prostate cancer | 90 | 12625 | 5.0901 |
| Gastric cancer | 22 | 7129 | 7.9247 |
| Colon cancer | 6 | 22283 | 3.0148 |
| OSCC | 27 | 22283 | 6.1938 |
| Renal cell carcinoma | 32 | 22283 | 4.6779 |

1. **Comparison of RankProd and t-test**

For each approach, we calculated the statistical power of identifying differentially expressed genes and the false discovery rate for different p-value cutoffs, based on 5 simulated datasets (Supplementary Figure 1).

**Supplementary Figure 1.** RankProd (*) has higher statistical power and lower FDR than the parametric approach (o).

1. **Bayesian mixture model for p-value distribution**

From the p-value distribution of the up-regulated, down-regulated, and the non-differentially expressed genes (Supplementary Figure 2), we can use a mixture beta model to estimate the proportion of genes in each of the three categories (simulated data has 54675 probesets, we used $p=q=0.05$ ; ):

**Supplementary Figure 2.** p-value distribution of the up-regulated, down-regulated, and the non-differentially expressed genes computed from the Rank Product one-sided test.

**Beta mixture model result**: In Supplementary Table 2, the left column represents the proportion of up-regulated genes that are simulated, and the right column shows the proportion estimated by the Beta mixture model. Ten simulations were performed for each proportion, and * represents the mean of the estimated proportion is significantly different (p < 0.005) from the true proportion. The estimation of down-regulated genes shows similar results.

**Supplementary Table 2**. The proportion of up-regulated genes simulated versus the proportion estimated by the Beta mixture model

| Proportion of up-regulated genes simulated | Proportion of up-regulated genes estimated |
| --- | --- |
| 0.01 | 0.051* |
| 0.02 | 0.055* |
| 0.03 | 0.055* |
| 0.04 | 0.058* |
| 0.05 | 0.061* |
| 0.06 | 0.067* |
| 0.07 | 0.073 |
| 0.08 | 0.079 |
| 0.09 | 0.087 |
| 0.1 | 0.093 |

Even if the beta-mixture model is not able to estimate the proportion of differentially expressed genes accurately, the CDEP approach can still achieve higher statistical power while maintaining low Type I error given different FDR values (Supplementary Table 3).

**Supplementary Table 3**. For low proportion of differentially expressed genes, CDEP approach is still more robust in obtaining higher statistical power while maintaining low Type I error rate

|  |  |  |  | CDEP | | Meta-Profile | | Meta-RankProd | |
| --- | --- | --- | --- | --- | --- | --- | --- | --- | --- |
| q | p | a4 | FDR | power | Type I error | power | Type I error | power | Type I error |
| 0.01 | 0.01 | 1 | 0.05 | 0.700 | 1.15x10^-4^ | 0.064 | 1.48x10^-5^ | 0.237 | 2.48x10^-3^ |
|  |  |  | 0.1 | 0.722 | 2.79x10^-4^ | 0.091 | 2.59x10^-5^ | 0.245 | 2.65x10^-3^ |
|  |  |  | 0.2 | 0.722 | 2.81x10^-4^ | 0.119 | 5.36x10^-5^ | 0.272 | 3.01x10^-3^ |
|  |  |  | 0.3 | 0.723 | 3.73x10^-4^ | 0.213 | 1.13x10^-4^ | 0.303 | 3.47x10^-3^ |

1. **Comparisons of different approaches for handling genes appearing in different numbers of datasets**

We categorized genes by the number of times they are differentially expressed among the 6 datasets, and evaluated the three meta-analysis methods' statistical power and type I error of detecting genes with consistently differential expression pattern. From the Suppl. Fig. 3 and 5, we observed that all methods obtain nearly 100% power for genes appearing in all 6 datasets, and this is not surprising as combining evidence across multiple datasets have been proved successful in these previous studies (Rhodes, et al., 2002; Hong, et al., 2008). For genes appearing only in 3-4 datasets, CDEP out performs the other two methods (except when FDR=0.01, a very stringent threshold for controlling multiple testing) while maintaining negligible type I error (Suppl. Fig. 4 and 6). We ascribe this improved performance to: i) the better estimation of false positives using the flexible beta-mixture model, and ii) the integration of the likelihood using different FDR values (*l*), which increases the sensitivity of the approach. Note that even though the Meta-RankProd article (Hong, et al., 2008) did not describe how to handle genes that do not appear in all datasets, we also used the median rank product for those genes in the samples where they are missing for the Meta-RankProd approach in order to compare fairly. For genes only appear in 1 or 2 datasets, both CDEP and Meta-Profile could only have zero power (Suppl. Figure 3 and 5) because the methods do not obtain enough information to suggest these genes are consistently differentially expressed (i.e. the combination of 1 or 2 strong signal with the median rank for 4 or 5 datasets is not enough for CDEP to call it as positives). As mentioned in the main text, the Meta-RankProd tends to be biased for calling a gene as positive if it is only differentially expressed in some of the datasets, particularly for the positive genes appearing in a dataset with larger number of samples. While the Meta-RankProd obtains higher power than CDEP and Meta-Profile, it tends to have higher Type I error than the other two approaches (Suppl. Figure 2 and 4).

We concluded that both CDEP and Meta-Profile are conservative approaches, while Meta-RankProd is a more vigorous method which could be biased on genes only differentially expressed in a subset of dataset. CDEP is shown to have higher statistical power for genes appearing in at least half of the datasets being studied when comparing with the other two approaches and it maintains low Type I error as comparable to Meta-Profile.


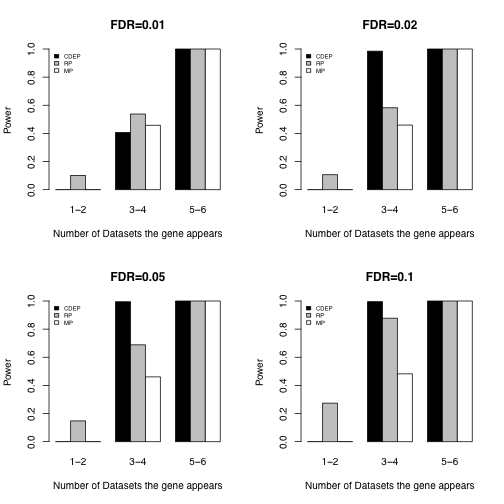


**Supplementary Figure 3.** The statistical power for identifying genes with consistent differential expression pattern for CDEP, Meta-RankProd, and Meta-Profile based on simulation. Different FDR criteria were used, and the x-axis on each plot shows the number of datasets a gene appears in.


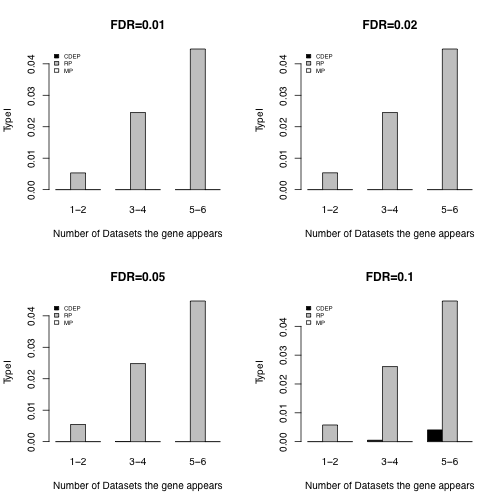


**Supplementary Figure 4**. The Type I error (falsely identified genes as consistently differentially expressed) for CDEP, Meta-RankProd, and Meta-Profile based on simulation. Different FDR criteria were used, and the x-axis on each plot shows the number of datasets a gene appears in.

**FDR=0.01**

**FDR=0.02**

**FDR=0.05**

**FDR=0.1**

**1-2 datasets**

**3-4 datasets**

**5-6 datasets**


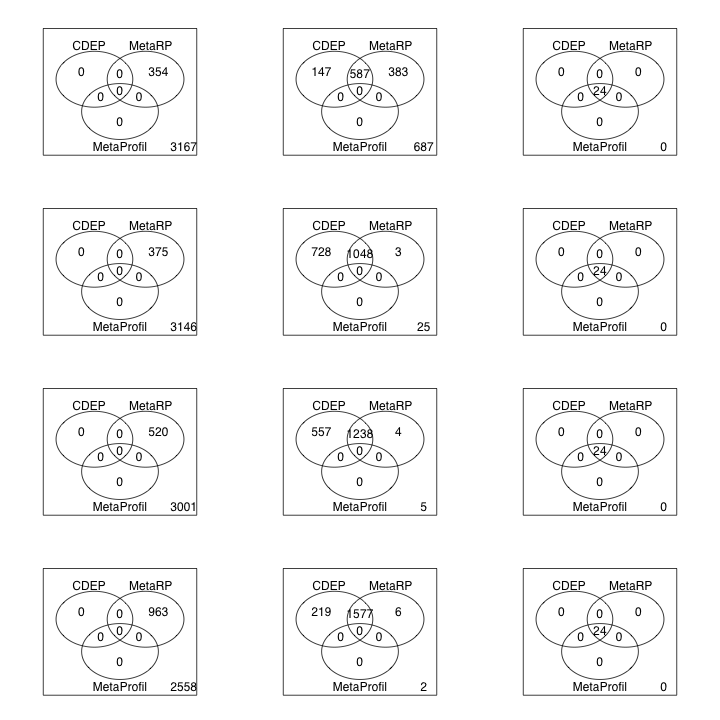


**Supplementary Figure 5.** The Venn diagram for genes with consistent differential expression pattern identified in the three approaches based on simulation. Rows represent different FDR criteria, and columns represent the number of datasets the genes appear in.

**FDR=0.01**

**FDR=0.02**

**FDR=0.05**

**FDR=0.1**

**1-2 datasets**

**3-4 datasets**

**5-6 datasets**


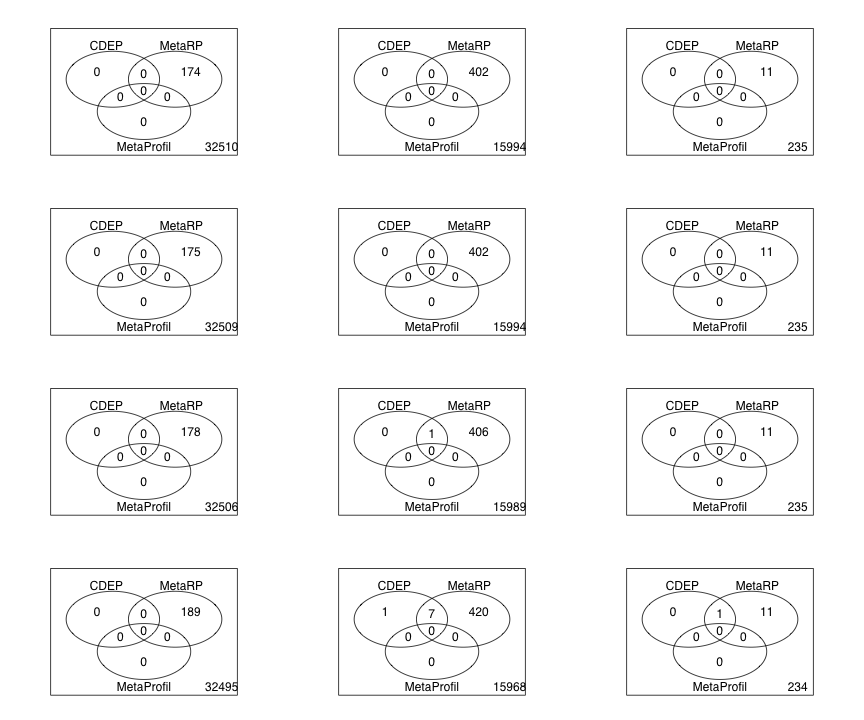


**Supplementary Figure 6.** The Venn diagram for genes falsely identified as consistently differentially expressed in the three approaches based on simulation. Rows represent different FDR criteria, and columns represent the number of datasets the genes appear in.

1. **Comparisons of the three approaches using the 6 cancer datasets as case study**

In addition to the comparisons made through simulation studies, we also used the 6 cancer datasets consisting of metastatic and primary cancer samples to compare CDEP with Meta-Profile and Meta-RankProd. At FDR=0.05, the results of these three approaches and the overlapping genes they identified are shown in Supplementary Figure 7. Specifically, for Meta-Profile, we first identified significant genes for each dataset, and then summarized the number of times each gene identified to be differentially expressed among the 6 datasets. We then permuted the statistics within each dataset while maintaining the proportion of differentially expressed genes. The permutation was used to estimate the FDR of observing the number of times the genes identified to be differentially expressed among the six datasets. For Meta-RankProd, the rank product statistic for each gene *g* was computed as: $RP_{g}=\left(\prod_{i} \prod_{k} r_{gik}\right)^{(1/K)}$, where *i* and *k* represent dataset and the within dataset pairwise comparison between the two conditions, respectively, and K is total number of pairwise comparison. Meta-RankProd then permuted the gene expression values within each array, and estimated the FDR for observing the rank product statistics. Since CDEP uses the median value of the statistic within each dataset for missing genes, we also applied this procedure for Meta-Profile and Meta-RankProd in order to achieve a fair comparison.

While Meta-Profile is an approach with high precision, it only identified a limited number of significant genes when FDR was set at 0.05, a commonly used standard threshold for such analysis (Even though the number of genes identified increased when increasing FDR, such practice ran the risk of introducing high number of false positives). This could be due to the fact that Meta-Profile is sensitive to the number of times a gene is identified as significant among the datasets used during meta-analysis, as Meta-Profile is based on the number of times a gene is identified as differentially expressed. Therefore, genes appearing only in a few datasets were unlikely to show up in the final results even if they are related to metastatic behavior. Other factors contributing to the reduced power include the limited number of datasets and the small number of samples within dataset for comparing the metastatic versus primary cancer.

On the other hand, Meta-RankProd tends to bias towards genes that are differentially expressed in a dataset with a large number of samples, as described in the Background section in the main text. The approach is suitable for meta-analysis combining homogeneous datasets (e.g. comparing metastatic versus primary cancer samples for the same cancer type but from different research groups). As our objective here is to identify core sets of genes related to metastatic behavior across different cancer types, Meta-RankProd would tend to identify more false positives (as shown in the simulation analysis) in this case study, an undesirable situation since false positives are very difficult to spot while using real data.

2

178

**CDEP**

**Meta-RankProd**

**Meta-Profile**

0

0

0

2787

59

**Supplementary Figure 7.** The Venn diagram shows the intersection of genes differentially expressed consistently in metastatic cancer samples as identified by CDEP, Meta-RankProd and Meta-Profile respectively. Six microarray datasets comparing metastatic versus primary cancer samples are used for this meta-analysis.
